# Metabolite Profiling of *Gardenia jasminoides* Ellis In Vitro Cultures with Different Levels of Differentiation

**DOI:** 10.3390/molecules27248906

**Published:** 2022-12-14

**Authors:** Gergana Krasteva, Strahil Berkov, Atanas Pavlov, Vasil Georgiev

**Affiliations:** 1 Laboratory of Cell Biosystems, Institute of Microbiology, Bulgarian Academy of Sciences, 139 Ruski Blvd., 4000 Plovdiv, Bulgaria; 2 Institute of Biodiversity and Ecosystem Research, Bulgarian Academy of Sciences, 23 Acad. G. Bonchev, 1113 Sofia, Bulgaria; 3 Department of Analytical Chemistry and Physical Chemistry, Technological Faculty, University of Food Technologies, 4002 Plovdiv, Bulgaria

**Keywords:** plant cell culture technology, antioxidants, phenolic, callus, shoots, cell suspension, somaclonal variation

## Abstract

*Gardenia jasminoides* Ellis is an aromatic and medicinal plant of high economic value. Much research has focused on the phytochemistry and biological activities of *Gardenia* fruit extracts; however, the potential of the *Gardenia* plant in vitro cultures used as mass production systems of valuable secondary metabolites has been understudied. This paper presents data on metabolite profiling (GC/MS and HPLC), antioxidant activities (DPPH, TEAC, FRAP, and CUPRAC), and SSR profiles of *G. jasminoides* plant leaves and in vitro cultures with different levels of differentiation (shoots, callus, and cell suspension). The data show strong correlations (r = 0.9777 to r = 0.9908) between antioxidant activity and the concentrations of chlorogenic acid, salicylic acid, rutin, and hesperidin. Eleven co-dominant microsatellite simple sequence repeats (SSRs) markers were used to evaluate genetic variations (average PIC = 0.738 ± 0.153). All of the investigated *Gardenia* in vitro cultures showed high genetic variabilities (average Na = 5.636 ± 2.157, average Ne = 3.0 ± 1.095). This is the first report on a study on metabolite profiles, antioxidant activities, and genetic variations of *G. jasminoides* in vitro cultures with different levels of differentiation.

## 1. Introduction

*Gardenia jasminoides* Ellis is a well-known medicinal plant of the Rubiaceae family, cultivated in China for over 2000 years. Dry fruits of the plant are listed as raw material in the Chinese and Japanese Pharmacopoeias, popularly as “Zhizi” in China, “Sanshishi” in Japan, and “Cape Jasmine” in Korea [1]. *G. jasminoides* alone or jointly with other substances (e.g., Zhi-Zi-Chi decoction, comprising *G. jasminoides* and *Semen sojae preparatum*), is widely used in traditional Chinese medicine for the treatment of several disorders [2,3]. Extracts from *Gardenia* fruits have strong antioxidant, anti-inflammatory, antidepressant, neuroprotective, anti-atherosclerotic, antithrombotic, antihypertensive, antitumor, antiangiogenic, and anti-diabetic activities [1,4,5,6,7,8,9]. Many types of research have focused on the metabolite profiling of *Gardenia* fruit extracts and some excellent review papers have been published on this topic [1,3,4,5,10,11,12,13]. The major constituents are iridoid glycosides, organic acids, flavonoids, saffron glycosides, phenolic acids, and triterpenoids. The most extensively studied substances are the saffron glycosides crocin and crocetin, which have strong neuroprotective, antidepressant, anti-hypertensive, anti-inflammatory, and nitric oxide inhibitory effects [3,9,14], and the iridoid glycosides geniposide and genipin, which have strong anti-inflammatory, antifungal, and melanogenesis inhibitory activity [15,16,17]. Crocin and crocetin are the major constituents of the popular natural water-soluble food colorant, known as “Gardenia yellow”. Genipin, a product from geniposide hydrolysis, is used as a natural biological crosslinking agent, and also as a blue colorant, known as “Gardenia blue” when linked with amino acids or protein hydrolysates [1]. It is worth noting that phenolic acids and flavonoids in *Gardenia* are less studied, even though they are considered to be responsible for the antioxidant activities of extracts [18,19], and have been associated with antiangiogenic activity [6]. Moreover, studies on the chemical composition of different organs of *Gardenia* plants (e.g., leaves) are scarce in the scientific literature [17,18], and studies on *Gardenia* in vitro cultures are almost missing [20].

Plant cell culture technology has been generally accepted as the most promising technology for the sustainable production of plant biomass and valuable secondary metabolites with the lowest eco-impact. Recently, many plant species have been cultivated in vitro on a commercial scale, mostly for the needs of the cosmetic, pharmaceutical, and food industries [21,22]. Since *G. jasminoides* is a species of great economic value not only for medicine and foods but also as an indoor and outdoor ornamental plant, many types of research have focused on its micropropagation [23,24,25,26,27,28,29,30]. Several factors, including culture medium [31,32], light [33,34], growth regulators [34,35], chemical mutagens [36,37], and explant encapsulation [38], have been studied for their effects on in vitro *Gardenia* propagation, plant regeneration, acclimatization, and induction of polymorphism. It was reported that growth regulators (e.g., 2iP) can induce somaclonal variation and formation of chimeric plants during in vitro stage of *Gardenia* micropropagation [35]. However, no genetic variation (analyzed by inter simple sequence repeat (ISSR) markers) was observed during micropropagation of apical shoots cultivated in a medium, supplied with 6-benzylaminopurine (BAP) [38]. Recently, the high efficiency of simple sequence repeat (SSR) markers for the detection of genetic variations in *Gardenia* was demonstrated during in vitro mutagenesis with Ethyl methanesulfonate (EMS) [36]. However, data on the initiation and characterization (metabolite profiling and genetic stability) of differentiated and undifferentiated in vitro cultures of *G. jasminoides* as potential secondary metabolite production systems are almost missing in the available literature [20].

This paper presents comparative metabolite profiling (GC/MS and HPLC) of *G. jasminoides* plant leaves and in vitro cultures with different levels of differentiation (shoots, callus, and cell suspension). Data were correlated with observed antioxidant activities (DPPH, TEAC, FRAP, and CUPRAC) and SSR profiles for the detection of genetic variabilities between the investigated plant and the corresponding in vitro systems. To our best knowledge, this is the first report on a complex study on metabolite profiles, antioxidant activities, and genetic variabilities of *G. jasminoides* in vitro cultures with different levels of differentiation. 

## 2. Results and Discussion 

### 2.1. GC/MS Profiling of Gardenia Plant Leaves and In Vitro Cultures

**Figure 1:**
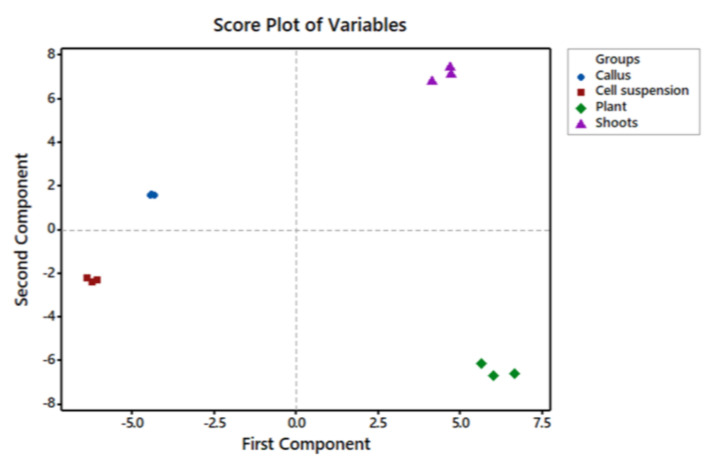
Score plot of the first two principal components identified by principal component analysis (PCA) of GC/MS identified metabolites in *G. jasminoides* plant leaves (green) and in vitro grown shoots (purple), callus (blue), and cell suspension (red) cultures.

The content of several groups of metabolites, such as hydrocarbons, fatty alcohols, fatty acids, organic acids, saccharides, terpenes, phytosterols, and free and bound phenolic acids has been identified in biomass of *G. jasminoides* plant leaves and in vitro grown shoot, callus, and cell suspension cultures by GC/MS. In total, 67 metabolites were identified in the plant and shoots, and 41 and 34 metabolites in callus and cell suspension cultures, respectively (Figures S2–S5, Table S1). Principal component analysis (PCA) showed that four principal components have eigenvalues greater than one, explaining 97.6% of the variation in the data. Figure 1 shows the score plot of the first and second principal components. PC1 has an eigenvalue of 31.641 and explains 40.1% of the variations. It has strong positive associations with Octadecane, 2-methyl; Tetracosane; 1-Eicosanol; Galactose; Galactosylglycerol; Sucrose; Protocatechuic acid; Quinic acid; *trans*-p-Hydroxycinnamic acid; 3,5-Dimethoxy-4-hydroxycinnamic acid and *trans*-Ferulic acid. PC2 has an eigenvalue of 27.415 and explains 34.7% of the variations. It has strong positive associations with Dodecanol; Heptane, branched (Hydrocarbon); Tetradecanoic acid (methyl ester C14:0); Hexadecanoic acid (methyl ester, Palmitic acid, C16:0); Octadecadienoic acid (methyl ester, Linoleic acid, C18:2); Octadecenoic acid (methyl ester, Oleic acid, C18:1); 1-Docosanol; 1-Tetracosanol; Octacosane;1-Octacosanol; Erythritol and Fructose. 

**Figure 2:**
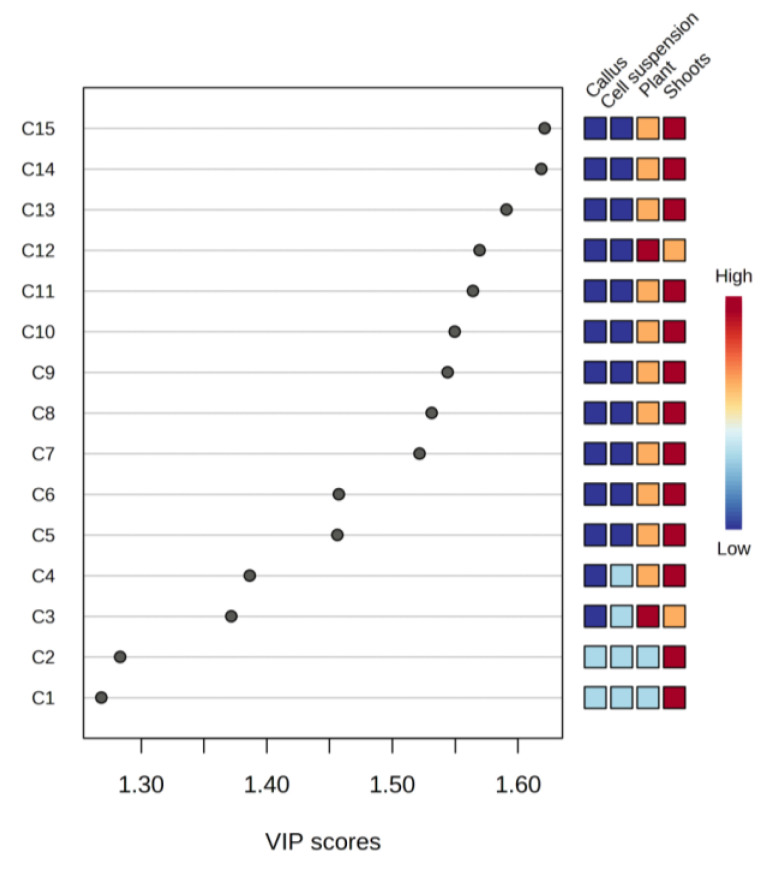
Variable importance in projection (VIP) scores plot (measure in PLS-DA), indicating the most discriminating compounds in descending order of importance: C1—Heptane, branched; C2—Tetradecanoic acid (methyl ester C14:0); C3—Sucrose; C4—Galactosylglycerol; C5—1-Hexacosanol; C6—2-Hydroxytricosanoic acid; C7—Arabitol; C8—Arabitol; C9—Eicosanoic acid (methyl ester, Arachidic acid, C20:0); C10—2-Hydroxy-hexacosanoic acid; C11—Octadecane, 2-methyl; C12—1-Eicosanol; C13—1-Octadecanol; C14—Hexacosane; C15—Tetracosane.

Partial least squares discriminant analysis (PLS-DA) was used to identify compounds that could discriminate between plant, shoot, callus, and cell suspension cultures. The variable importance in projection (VIP) scores of discriminating compounds is presented in Figure 2. Interestingly, hydrocarbons and fatty alcohols such as Tetracosane, Hexacosane; and 1- Octadecanol are present in the highest concentrations in shoot cultures, lower in the plant, and completely absent in the callus, and cell suspensions. In contrast, in *Pancratium maritimum* L. (a monocot species), hydrocarbons were more abundant in the undifferentiated callus culture, whereas fatty alcohols were found in plant leaves and differentiated shoot cultures [39].

**Figure 3:**
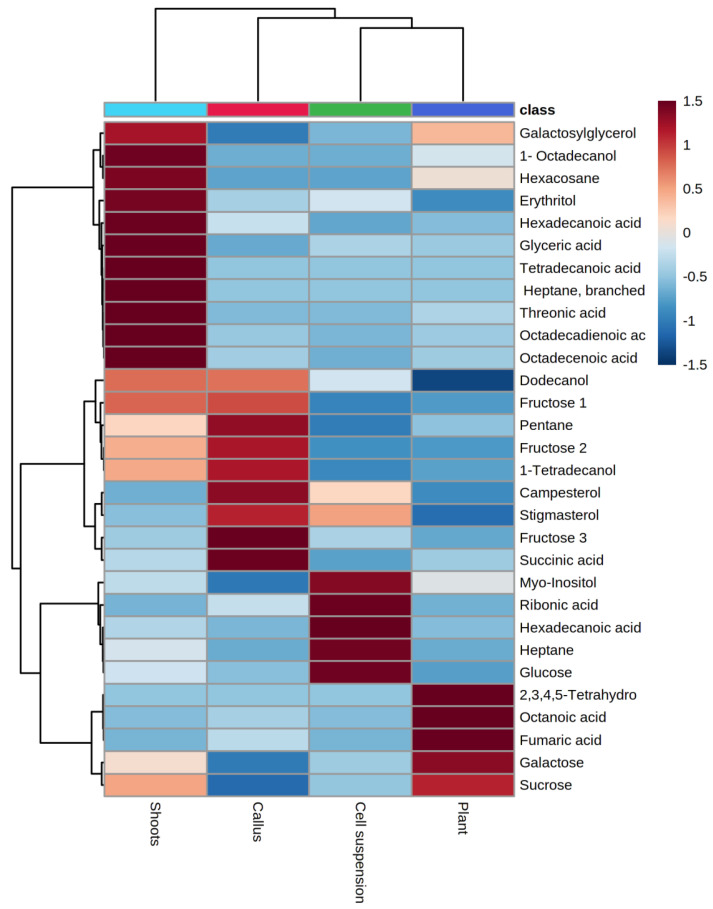
Hierarchically clustered heatmap of 30 most significant (T-test/ANOVA, *n* = 3) metabolites found in *G. jasminoides* plant leaves (Purple) and in vitro grown shoots (light blue), callus (red), and cell suspension (green) cultures, identified and quantified by GC/MS. Clustering was performed by using the Ward method with Euclidean distance. Each column in the Heatmap analysis represents a sample (plant, shoots, callus, and cell suspensions), and each row indicates metabolite concentrations at their highest and lowest levels (ranging from +1.5 to −1.5), coded in red and blue colors with different intensities.

For better visualization of the differences observed in the metabolite patterns of the investigated samples, the relative concentrations of 30 most significantly different metabolites identified in *Gardenia* plant leaves, shoot, callus, and cell suspension cultures (determined by processing the GC/MS data with ANOVA, *t*-test) were mapped in a hierarchically clustered heatmap (Figure 3). The clustering of the groups indicated a clear and strong difference between the shoot culture and the other samples (callus, cell suspension, and plant leaves). The most abundant metabolites found in the shoots were fatty acids, hydrocarbons, and fatty alcohols. The callus culture was rich in phytosterols and saccharides. The cell suspension was rich in sugars and sugar acids, whereas sugars, fatty, and organic acids predominated in plant leaves. 

### 2.2. HPLC Quantification of Phenolic Compounds in Gardenia Plant Leaves and In Vitro Cultures

It is well known that plant secondary metabolites, phenolics, in particular, have multiple biological activities [40]. In this study, we analyzed the phenolic content (nine phenolic acids and six flavonoids) of *G. jasminoides* plant leaves and in vitro cultivated shoot, callus, and cell suspension cultures (Table 1). As expected, the samples of differentiated tissues (plant leaves and shoots) accumulated a more diverse mix of phenolics, and in higher concentrations than those found in undifferentiated tissues (callus and cell suspension). However, there are some interesting differences. Analyses of data showed that shoot cultures accumulated chlorogenic acid, quercetin, kaempferol, and rutin in significantly higher (*p* < 0.01) amounts when compared to plant leaves (Table 1, Figure S7). Moreover, shoot cultures were the only samples that accumulated protocatechuic and p-coumaric acids. Rutin is one of the major flavonoids reported in *Gardenia* plant extracts (mostly in fruits) [1,4,10,11,18], and in this study, it was shown to accumulate in higher amounts in differentiated than in undifferentiated *Gardenia* in vitro cultures. 

It is worth noting that undifferentiated callus and cell suspension cultures were the only in vitro systems that produced (+)-catechin, and also accumulated vanillic acid in contrast to plant leaves (Table 1, Figures S8 and S9). Recently, it was demonstrated that in vitro cultivated *Gardenia* calli and cell suspensions can produce significant amounts of chlorogenic acid derivatives [20]. The authors further improved cell suspension productivity and achieved a yield of total chlorogenic acid derivatives as high as 20.98 mg/g DW by using methyl jasmonate as an elicitor. Here, a higher amount of chlorogenic acid was also found in the cell suspension culture compared to the callus culture, which suggests that cell suspensions of *Gardenia jasminoides* could be used as a promising source in the production of chlorogenic acid and its derivatives. A recent study based on molecular docking showed that chlorogenic acid can interact with α-glucosidase and α-amylase and could be considered as potential candidate molecules with anti-diabetic activity found in methanolic extracts from *Gardenia* [10]. 

### 2.3. Antioxidant Activities of Phenolic Extracts from Gardenia Plant Leaves and In Vitro Cultures

The strong antioxidant activities of *Gardenia* plant extracts have been reported in several studies [18,19,41]. Recently, it was clearly demonstrated that the major antioxidants in *Gardenia* plant extracts were not crocins or geniposides, but phenolics [12]. In this study, we analyzed the antioxidant activities of methanol extracts from *G. jasminoides* plant leaves, shoot, callus, and cell suspension cultures by using four reliable in vitro assays: DPPH, TEAC, FRAP, and CUPRAC (Table 2). The extract from plant leaves showed the highest total phenolic content (4.05 ± 0.36 mg GAE/g DW), followed by the extracts from shoot, cell suspension, and callus cultures (Table 2). The antioxidant potentials of the extracts from undifferentiated in vitro cultures (callus and cell suspension) were significantly lower (*p* < 0.01) when compared to plant leaves and differentiated shoots (Table 2). Moreover, the extracts from cell suspension and callus cultures showed higher potential to reduce cupric ions (CUPRAC values from 53.45 ± 8.41 and 49.56 ± 8.50 mM TE/g DW, respectively), followed by the ability to scavenge ABTS radicals, DPPH radicals, and to reduce ferric ions (Table 2). Interestingly, the extract from the differentiated shoot culture showed the highest antioxidant activity against DPPH radicals (647.21 ± 33.29) among all investigated extracts. Debnath et al. reported that water extract of *G. jasminoides* fruit (rich in phenolics and flavonoids) showed strong DPPH radical scavenging activity, followed by ABTS radical scavenging activity, and ferric ion reducing capacity [19]. The authors observed a high correlation between the antioxidant capacity and the total phenolic and total flavonoid contents of the extract. It has been reported that the extract of *G. jasminoides* leaves (containing gallic acid, (+)-catechin, rutin hydrate, and quercetin) exhibits antioxidant activity comparable to that of vitamin C, which confirms the role of phenolics and flavonoids as the main contributors to that effect [18]. However, no correlation analysis of the relationship between the concentration of individual compounds and antioxidant activities was reported. 

The correlation analysis of our data showed the existence of a strong correlations between (1) the ability of extracts to scavenge the DPPH radical and the concentrations of chlorogenic acids, salicylic acid, and rutin (r = 0.9892; *p* = 1.13 × 10^−9^; r = 0.9777; *p* = 4.18 × 10^−8^ and r = 0.9807; *p* = 2.05 × 10^−8^); (2) the ability of extracts to scavenge the ABTS radical and the concentrations of salicylic acids, rutin, and hesperidin (r = 0.9899; *p* = 8.26 × 10^−10^; r = 0.9854; *p* = 5.12 × 10^−9^ and r = 0.9908; *p* = 5.15 × 10^−10^); (3) the ability of extracts to reduce ferric ions and the concentrations of salicylic acids, rutin, and hesperidin (r = 0.9868; *p* = 3.07 × 10^−9^; r = 0.9864; *p* = 3.59 × 10^−9^ and r = 0.9843; *p* = 7.37 × 10^−9^); and (4) the ability of extracts to reduce cupric ions and the concentrations of salicylic acids, rutin, and hesperidin (r = 0.9821; *p* = 1.41 × 10^−8^; r = 0.9792; *p* = 2.95 × 10^−8^ and r = 0.9862; *p* = 3.81 × 10^−9^) (Tables S2 and S3). Based on the correlation analyses, it can be suggested that chlorogenic acid, salicylic acid, rutin, and hesperidin were the most active antioxidants in methanol extracts from *G. jasminoides* plant leaves, shoot, callus, and cell suspension cultures. Data obtained in this study are in agreement with previous reports that suggested the role of chlorogenic acid derivatives and rutin as the major antioxidants in *Gardenia* [18,20].

### 2.4. Somaclonal Variation in Gardenia In Vitro Cultures

The genetic diversity of differentiated (shoots) and undifferentiated (callus and cell suspension) *Gardenia* in vitro cultures was evaluated by using co-dominant microsatellite simple sequence repeat (SSR) markers. Eleven SSR markers developed for *Gardenia* by Xu et al. [42] and Deng et al. [43] were selected for this study on the basis of their polymorphic information content (PIC). Data showed that all SSRs, except GJ08, have more than three alleles and can be used for the evaluation of genetic diversity in the investigated *Gardenia* systems (Table 3). The GJ08 marker also showed the lowest polymorphic information content (PIC = 0.375), which makes it ineffective for the evaluation of genetic diversity. The number of detected alleles (Na) ranged from 2 to 6 with an average of 5.636 ± 2.157 alleles per locus, whereas the effective number of alleles (Ne) ranged from 1 to 4 with an average of 3.0 ± 1.095. These values were significantly higher than those reported for micropropagated *Gardenia* somaclones (average Na = 1.85, average Ne = 1.85) [36], which showed that the differentiated and undifferentiated *Gardenia* in vitro cultures used in this study have much higher genetic variabilities. This was also confirmed by the high values of observed heterozygosity (Ho = 1) and expected heterozygosity (He = 0.972 ± 0.051), which is clear evidence of high genetic variability among the investigated systems.

**Figure 4:**
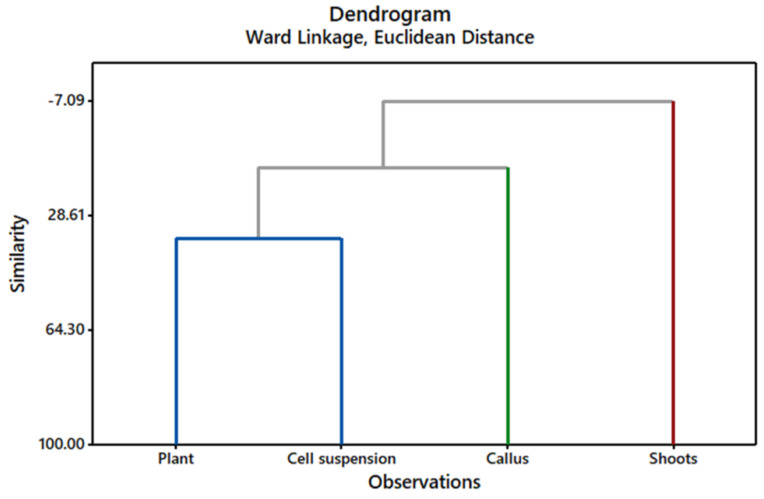
Dendrogram constructed using SSR data and Ward’s clustering method with Euclidean distance. The dendrogram shows genetic similarities between *Gardenia* plant, differentiated shoots and undifferentiated callus, and cell suspension cultures. Cultures having different genetic similarities were grouped in clusters with different colors (cluster 1—blue, cluster 2—green, and cluster 3—red).

Hierarchical cluster analysis of SSR data grouped the *Gardenia* plant and its in vitro cultures into clusters by using Euclidean distance similarity coefficients (Figure 4). The highest genetic similarity (35.47) was found between the plant and cell suspension culture, whereas the similarities between plant and callus (13.53) and plant and shoots (−7.09) were significantly lower (Figure 4). Interestingly, a similar pattern was observed when clustering GC/MS data for the cultures (Figure 3), where the highest similarity (17.91) was observed between the plant and cell suspension, followed by plant and callus (12.84), and the lowest similarity was between plant and shoots (3.75). Analyzing the data, it can be speculated that the significant differences observed in the metabolite profile of the differentiated shoot culture correlated with the observed high genetic distance when compared to the *Gardenia* plant and undifferentiated in vitro cultures (calli and cell suspensions). This is an interesting observation since it is generally postulated that differentiated in vitro cultures show less genetic variability than undifferentiated ones [44]. 

## 3. Materials and Methods

### 3.1. Plant Material

*Gardenia jasminoides* Ellis plant was purchased from a certified international nursery with all necessary certificates for plant identity. The shoot cultures were derived from sterilized nodal segments, and transferred on MS medium with the addition of 30.0 g/L sucrose, 4.0 mg/L BAP and 5% plant-agar (Duchefa Biochemie, Haarlem, The Netherlands). Callus cultures were initiated from sterilized young leaves, transferred on half-strength MS medium, supplemented with 30.0 g/L sucrose, 2.0 mg/L NAA, 0.5 mg/L BAP, and 5% plant-agar. The cell suspension culture was initiated by transferring callus aggregates into a liquid half-strength MS medium with the same composition, and cultivation on an orbital shaker (110 rpm) with sub-cultivation periods of 21 days to obtain a stable homogeneous suspension culture.

For the experiments, fully developed plant leaves and 21-day-old biomass of in vitro cultures were used. The biomass was frozen and freeze-dried (Christ Alpha 1-2, Martin Christ, Osterode am Harz, Germany) before being used in experiments.

### 3.2. GC/MS Analyses

Extraction of biomass for GC/MS analysis was performed according to Nikolova et al. 2019 [45]. Briefly, 100 mg of dried biomass was supplemented with internal standards and extracted with 1 mL methanol for 24 h. Then, 800 µL of the extract was mixed with 500 µL distillated water and 500 µL of chloroform. After mixing and centrifuging, the chloroform fraction was separated, evaporated, and transmethylated with 2% sulfuric acid in methanol. Lipids were extracted with n-hexane, which was evaporated to obtain the lipid fraction, and 100 µL of the aqueous fraction was evaporated to obtain the polar fraction. The rest of the aqueous fraction was hydrolyzed with sodium hydroxide and then acidified with hydrochloric acid. The phenolic compounds were extracted with ethyl acetate, which were dried and evaporated to obtain phenolic fraction. The biomass remaining after methanol extraction was subsequently hydrolyzed first with 2 M sodium hydroxide followed by acid hydrolysis with 6 M hydrochloric acid to obtain two fractions of methanol insoluble-bound alkaline and acid hydrolyzable phenolic acids, respectively. The extractions and fractions obtained were silylated according to Berkov et al. [46].

The extracts were analyzed on a Thermo Scientific Focus GC coupled with a Thermo Scientific DSQ mass detector. The operation mode was in EI at 70 eV, and an ADB-5MS column (30 m × 0.25 mm × 0.25 μm) was used. The injector temperature was 250 °C. The temperature program was: 100–180 °C at 15 °C/min, 180–300 °C at 5 °C/min, and 10 min hold at 300 °C. 0.8 mL/min flow rate of carrier gas (Helium) was used. The injection volume of 1 µL at a split ratio of 1:10 was applied. The metabolites were identified as TMSi derivatives by comparing their mass spectra and Kovats Indexes (RI) with commercial and online available plant-specific databases (The Golm Metabolome Database http://gmd.mpimp-golm.mpg.de, accessed on 06 June 2022/; NIST2011; WILEY2009; FAMES2011). The measured mass spectra were deconvoluted by the Automated Mass Spectral Deconvolution and Identification System (AMDIS). RI of the compounds was recorded with standard n-hydrocarbon calibration mixture (C9–C36) (Restek, Cat no. 31614, supplied by Teknokroma, Barcelona, Spain) using AMDIS 3.6 software [45].

### 3.3. HPLC Analyses

Approximately 500 mg of freeze-dried biomass of plant leaves, and 21-day-old shoots, callus, and cell suspension cultures were extracted in triplicate with 3 × 10 mL 70% methanol in an ultrasonic bath for 30 min. After filtration, the methanol was evaporated (45 °C, under vacuum) and the water fraction was adjusted with distilled water to 50 mL in a volumetric flask; 20 mL of this extract was subjected to solid-phase extraction by using Strata C18-E (55 um, 70A; 500 mg/6 mL) SPE cartridges (Phenomenex Inc., Torrance, CA, USA), preconditioned according to the manufacturer’s manual. The polar fractions were removed with 20 mL of distilled water, and the phenolic fraction was eluted with 2 mL methanol. This fraction was subjected to subsequent HPLC quantification and analyses of antioxidant activities.

The extracts obtained were analyzed by High-performance liquid chromatography (HPLC) as described previously [47]. The HPLC system consisted of a Waters 1525 Binary Pump (Waters, Milford, MA, USA), equipped with a Waters 2484 dual λ Absorbance Detector (Waters, Milford, MA, USA) and a Supelco Discovery HS C18 column (5 µm, 25 cm × 4.6 mm). The sample injection volume was 20 µL, and a flow rate of 1.0 mL/min was used. Gradient elution with 1% acetic acid in water (Solvent A) and methanol (Solvent B) with the following change for Solvent A was applied: 0 to 36 min Solvent A decreased from 90% to 78%; 36 to 37 min decrease from 78% to 70%; 37 to 47 min decrease from 70% to 60%; 47 to 58 min decrease from 60% to 54%; 58 to 59 min decrease from 54% to 40%; 59 to 71 min decrease from 40% to 20%; 71 to 72 min increase from 20% to 90%; 72 to 75 min hold to 90%. The detection wavelength was 280 nm for gallic acid, protocatechuic acid, (+)-catechin, vanillic acid, syringic acid, (-)-epicatechin, p-coumaric acid, salicylic acid, hesperidin, and 360 nm for chlorogenic acid, caffeic acid, ferulic acid, rutin, rosmarinic acid, quercetin, and kaempferol. For compound quantification, calibration curves built with standard compounds in concentrations of 10, 15, 25, 50, 100, 200, and 500 µg/mL were used. 

### 3.4. Antioxidant Activity Analyses

The antioxidant activities of methanol extracts, obtained as described in Section 3.2., were analyzed by using DPPH, TEAC (Trolox equivalent antioxidant capacity), FRAP (Ferric reducing antioxidant power), and CUPRAC (Cupric ion reducing antioxidant capacity) assays, following the previously described procedures [48] modified as follow:

#### 3.4.1. DPPH Assay

The investigated extract (20 µL) was mixed with 280 µL 0.1 mM solution of 1,1-Diphenyl-2-picrylhydrazyl radical (DPPH) in methanol in a 96-well plate by using Thermo Scientific E1-ClipTip Electronic Multichannel Pipette (8 technical replicates per single sample). A blank sample was developed in the same way, but 20 µL of methanol was added instead of extract. The plate was loaded into a microplate photometer (Multiskan FC, Thermo Scientific) and incubated at 37 °C with shaking for 15 min, followed by measurement of absorbance at λ = 515 nm. Standard solutions of 6-hydroxy-2,5,7,8-tetramethylchroman-2-carboxylic acid (Trolox), in concentrations of 50, 100, 200, 250, 300, and 500 µM were used to build a calibration curve (%DPPH inhibition vs. concentration; r^2^ = 0.9924). The antioxidant activity was expressed as µM Trolox equivalents (TE) per gram of dry biomass.

#### 3.4.2. TEAC Assay

The investigated extract (20 µL) was mixed with 280 µL ABTS radical (generated by mixing aliquots of 7 mM 2,2′azinobis (3)-ethylbenzthiazoline-6-sulfonic acid and 2.45 mM potassium persulfate for 16 h in darkness). A blank sample was developed in the same way, but 20 µL of methanol was added instead of extract. The plate was loaded into a microplate photometer (Multiskan FC, Thermo Scientific) and incubated at 37 °C with shaking for 15 min, followed by measurement of absorbance at λ = 734 nm. Standard solutions of Trolox, in concentrations of 50, 100, 200, 250, and 300 µM were used to build a calibration curve (%ABTS inhibition vs. concentration; r^2^ = 0.9943). The antioxidant activity was expressed as µM Trolox equivalents (TE) per gram of dry biomass.

#### 3.4.3. FRAP Assay

The investigated extract (20 µL) was mixed with 280 µL freshly prepared FRAP reagent (10 parts of 300 mM sodium acetate buffer with pH 3.6; 1 part of 10 mM 2,4,6 tripyridyl-s-triazine in 40 mM hydrochloric acid and 1 part of 20 mM iron(III) chloride hexahydrate in water). A blank sample was developed in the same way, but 20 µL of methanol was added instead of extract. The plate was loaded into a microplate photometer (Multiskan FC, Thermo Scientific) and incubated at 37 °C with shaking for 10 min, followed by measurement of absorbance at λ = 593 nm against the blank. Standard solutions of Trolox, in concentrations of 20, 40, 80, 120, and 200 µM were used to build a calibration curve (absorption vs. concentration; r^2^ = 0.9976). The antioxidant activity was expressed as µM Trolox equivalents (TE) per gram of dry biomass.

#### 3.4.4. CUPRAC Assay

The investigated extract (20 µL) was mixed with 70 µL 10 mM copper dichloride hydrate, 70 µL 7.5 mM neocuproine, 70 µL 1 M ammonium acetate buffer (pH 7.0), and 70 µL distilled water. A blank sample was developed in the same way, but 20 µL of methanol was added instead of extract. The plate was loaded into a microplate photometer (Multiskan FC, Thermo Scientific) and incubated at 37 °C with shaking for 10 min, followed by measurement of absorbance at λ = 450 nm against the blank. Standard solutions of Trolox, in concentrations of 20, 40, 80, 120, and 200 µM were used to build a calibration curve (absorption vs. concentration; r^2^ = 0.9912). The antioxidant activity was expressed as µM Trolox equivalents (TE) per gram of dry biomass.

#### 3.4.5. Total Phenolic Assay

The analyses of total phenolic content were performed by using Folin–Ciocalteu reagent. The investigated extract (20 µL) was mixed with 180 µL Folin–Ciocalteu reagent (10× diluted). The plate was mixed for 2 min and then 100 µL 7.5% sodium carbonate was added. A blank sample was developed in the same way, but 20 µL of methanol was added instead of extract. The plate was loaded into a microplate photometer (Multiskan FC, Thermo Scientific) and incubated at 37 °C with shaking for 8 min, followed by measurement of absorbance at λ = 750 nm against the blank. Standard solutions of gallic acid, in concentrations of 5, 10, 20, 30, 50, 100, 200, and 300 mg/L were used to build a calibration curve (absorption vs. concentration; r^2^ = 0.9969). The antioxidant activity was expressed as mg gallic acid equivalents (GAE) per gram of dry biomass.

### 3.5. Analyses of Somaclonal Variation

#### 3.5.1. DNA Extraction

Total DNA was extracted from 100 mg fresh biomass by using the DNeasy Plant Pro Kit (Qiagen, cat# 69204) according to the manufacturer’s instructions. The quantity and quality of DNA were measured spectrophotometrically (Biochrom WPA Biowave DNA, operated with BioDrop 125 CUVETTE) by measuring 260/280 and 260/230 ratios, and the integrity was checked by electrophoresis (1% agarose). The final concentration of DNA was adjusted to 15 ng/µL.

#### 3.5.2. SSR Analysis

Eleven SSR markers, reported previously by Xu et al. [42] and Deng et al. [43] were selected for this study (Table S4). For PCR amplification, GoTaq Green Master Mix (Promega, cat.# M7122) was used according to the kit’s manual. The reaction mixture (25 µL) consisted of 12.5 µL GoTaq Green Master Mix 2×, 5.0 µL DNA template, 2.5 µL forward primer, 2.5 µL reverse primer, and 2.5 µL nuclease-free water. Amplification was performed on a thermal cycler (Peqlab Primus 25 advanced) using the following program: initial DNA denaturation (2 min at 95 °C), followed by 35 cycles of denaturation (1 min at 95 °C), annealing (1 min at 50 °C), and polymerization (3 min at 72 °C), and final extension step at 72 °C for 10 min. The PCR products were resolved in 2% agarose gels (Bio-Rad PowerPac HC), and the bands were detected and quantified by using Bio-Rad Image Lab 6.0.1 software. 

### 3.6. Statistical Analyses

In all experiments, three independent biological samples were analyzed (*n* = 3). All spectrophotometric experiments were carried out in 8 technical repeats. The results are expressed as mean values (*n* = 3) with standard deviations (±SD). The means were statistically compared using one-way ANOVA, with Dunnett’s post hoc test to compare the means with the control. The Tukey method was used to compare the means. The differences between the means were considered significant for values of *p* ≤ 0.01. Correlation analyses of the data were performed by using the Pearson correlation method. Data were normalized by using normalization factors = 1 and Log 10 data transformation. The normality test was performed for each group of variables. Statistical tests were performed by using Metaboanalyst 5.0 (https://www.metaboanalyst.ca/ accessed on 3 October 2022) and MiniTab 17 Statistical Software (Minitab INC, State College, PA, USA). The number of alleles per locus (Na), effective number of alleles (Ne), observed heterozygosity (Ho), expected heterozygosity (He), and polymorphic information content (PIC) were estimated by using the Cervus 3.0.7 software package (http://www.fieldgenetics.com/ accessed on 12 September 2022). Subsequently, a genetic similarity dendrogram was constructed employing the Ward method with Euclidean distance.

## 4. Conclusions

In conclusion, our results clearly demonstrated the existence of significant differences in the metabolite profiles (both GC/MS and HPLC) of *G. jasminoides* plant leaves, differentiated shoots, and undifferentiated callus and cell suspension cultures. Shoot cultures accumulated phenolics in comparable or higher concentrations to that, found in plant leaves, whereas callus and cell suspension cultures showed significantly lower biosynthetic potential. All types of in vitro cultures produced compounds not detectable in plant leaves (e.g., protocatechuic and p-coumaric acids were found in shoots, and (+)—catechin was found in callus and cell suspension). Data in this study showed the existence of strong correlations between the concentrations of chlorogenic acid, salicylic acid, rutin, and hesperidin and the antioxidant activities of *G. jasminoides* methanol extracts. These results support the previously formulated suggestions that chlorogenic acid and rutin are the major antioxidants found in *Gardenia* extracts. SSR analyses revealed the existence of high genetic variability among investigated differentiated and undifferentiated *Gardenia* in vitro cultures. Based on clustering analyses of SSR and GC/MS data, we can speculate that the observed differences in primary and secondary metabolites, found in differentiated and undifferentiated *G. jasminoides* in vitro culture, could be closely related to the genetic variabilities of the cultures. However, more experiments have to be conducted to prove this speculation. 

## Figures and Tables

**Table 1:** HPLC analyses of phenolics found in *G. jasminoides* plant leaves, in vitro shoot, callus, and cell suspension cultures.

Compounds	Plant, µg/g DW	Shoots, µg/g DW	Callus, µg/g DW	Cell Suspension, µg/g DW
Protocatechuic acid	ND ^b,^*	8.65 ± 0.41 ^a^	ND ^b^	ND ^b^
(+)-Catechin	ND ^c^	ND ^c^	28.24 ± 0.99 ^b^	47.13 ± 3.40 ^a^
Chlorogenic acid	173.02 ± 9.67 ^b^	411.47 ± 11.67 ^a^	10.21 ± 0.77 ^c^	12.08 ± 0.27 ^c^
Vanillic acid	ND ^c^	375.02 ± 19.34 ^a^	16.21 ± 1.03 ^b^	17.48 ± 1.38 ^b^
Caffeic acid	84.34 ± 9.16 ^a^	97.57 ± 7.12 ^a^	28.32 ± 0.95 ^b^	ND ^c^
Syringic acid	84.68 ± 1.34 ^a^	26.85 ± 1.58 ^b^	16.98 ± 0.01 ^c^	14.01 ± 2.96 ^c^
(−)-Epicatechin	386.83 ± 10.90 ^a^	177.86 ± 4.53 ^b^	48.44 ± 1.84 ^c^	53.31 ± 2.86 ^c^
p-Coumaric acid	ND ^b^	15.15 ± 2.52 ^a^	ND ^b^	ND ^b^
Ferulic acid	83.38 ± 9.93 ^a^	32.64 ± 3.28 ^b^	21.76 ± 2.40 ^bc^	13.74 ± 1.32 ^c^
Salicylic acid	1344.97 ± 100.11 ^a^	1262.82 ± 193.99 ^a^	10.67 ± 3.37 ^b^	9.78 ± 1.09 ^b^
Rutin	40.75 ± 1.50 ^a^	50.35 ± 9.59 ^a^	3.18 ± 0.40 ^b^	3.06 ± 0.07 ^b^
Hesperidin	309.32 ± 13.40 ^a^	233.42 ± 46.31 ^b^	ND ^c^	ND ^c^
Rosmarinic acid	1636.22 ± 135.45 ^a^	419.72 ± 101.73 ^b^	31.27 ± 6.50 ^c^	32.30 ± 1.98 ^c^
Quercetin	39.33 ± 2.45 ^b^	284.28 ± 22.85 ^a^	0.12 ± 0.02 ^c^	ND ^c^
Kaempferol	4.15 ± 1.63 ^b^	286.05 ± 24.56 ^a^	ND ^c^	ND ^c^

* ND—Not Detected. The presented values are Means ± SD, *n* = 3. Means that do not share a letter are significantly different (*p* < 0.01, ANOVA with Tukey pairwise comparisons of means).

**Table 2:** Antioxidant activities of methanol extracts of *G. jasminoides* plant leaves, in vitro shoot, callus, and cell suspension cultures.

Assay	Plant	Shoots	Callus	Cell Suspension
Total Phenolics, mg GAE/gDW	4.05 ± 0.36 ^a^	3.03 ± 0.20 ^b,^*	0.18 ± 0.02 ^c,^*	0.27 ± 0.01 ^c,^*
DPPH, µM TE/g DW	368.41 ± 41.77 ^b^	647.21 ± 33.29 ^a,^*	8.73 ± 1.50 ^c,^*	18.29 ± 2.69 ^c,^*
TEAC, µM TE/g DW	2162.79 ± 62.62 ^a^	1394.90 ± 15.00 ^b,^*	12.50 ± 1.72 ^c,^*	23.57 ± 2.09 ^c,^*
FRAP, µM TE/g DW	251.68 ± 16.45 ^a^	234.56 ± 15.59 ^a,^*	5.47 ± 0.65 ^b,^*	10.95 ± 0.50 ^b,^*
CUPRAC, µM TE/g DW	349.74 ± 39.39 ^a^	281.28 ± 17.34 ^b,^*	49.56 ± 8.50 ^c,^*	53.45 ± 8.41 ^c,^*

The presented values are Means ± SD, *n* = 3. Mean values marked with “*” in rows are significantly different (*p* <  0.01, ANOVA with Dunnett’s post hoc test) from the control (Plant leaves). Means that do not share a letter are significantly different (*p* < 0.01, ANOVA with Tukey pairwise comparisons of means).

**Table 3:** Number of detected alleles (Na), effective number of alleles (Ne), observed heterozygosity (Ho), expected heterozygosity (He), and polymorphic information content (PIC) observed with 11 microsatellite loci used in this study.

SSR Locus	Na	Ne	Ho	He	PIC
GJ02	3	2	1	0.833	0.555
GJ03	5	3	1	0.933	0.744
GJ04	4	2	1	1	0.703
GJ08	2	1	1	1	0.375
GJ09	8	4	1	1	0.861
GJ10	8	4	1	1	0.861
GJ16	7	4	1	0.964	0.825
GJ17	4	2	1	1	0.703
eGJ010	7	4	1	0.964	0.825
eGJ118	8	4	1	1	0.861
eGJ144	6	3	1	1	0.810
mean ± SD	5.636 ± 2.157	3.0 ± 1.095	1.0 ± 0.0	0.972 ± 0.051	0.738 ± 0.153

## Data Availability

All data used to support the findings of this study are included within the article and Supplementary Materials.

## Supplementary Materials

The following supporting information can be downloaded at: https://www.mdpi.com/article/10.3390/molecules27248906/s1, Figure S1. *G. jasminoides* plant (A) and 21-day-old in vitro shoots (B), callus (C), and cell suspension (D) cultures; Figure S2. GC/MS chromatogram of extract of *G. jasminoides* plant leaves; Figure S3. GC/MS chromatogram of extract of *G. jasminoides* shoots culture; Figure S4. GC/MS chromatogram of extract of *G. jasminoides* callus culture; Figure S5. GC/MS chromatogram of extract of *G. jasminoides* cell suspension culture; Figure S6. GC/MS data before and after normalization (applied normalization factors = 1, Log 10 data transformation); Figure S7. Volcano plot of HPLC data for phenolics found in *G. jasminoides* in vitro shoot culture in comparison with the same, found in plant leaves; Figure S8. Volcano plot of HPLC data for phenolics found in *G. jasminoides* in vitro callus culture in comparison with the same, found in plant leaves; Figure S9. Volcano plot of HPLC data for phenolics found in *G. jasminoides* in vitro cell suspension culture in comparison with the same, found in plant leaves; Table S1. Metabolites identified in extracts of *G. jasminoides* plant leaves and in vitro grown shoots, callus, and cell suspension by GC/MS; Table S2. Pearson correlation coefficients (r) between observed antioxidant activities (DPPH, TEAC, FRAP, and CUPRAC) and the concentrations of phenolic compounds detected in extracts of *G. jasminoides* plant leaves and in vitro grown shoots, callus, and cell suspension; Table S3. *p*-values of calculated Pearson correlations between observed antioxidant activities (DPPH, TEAC, FRAP, and CUPRAC) and the concentrations of phenolic compounds detected in extracts of *G. jasminoides* plant leaves and in vitro grown shoots, callus, and cell suspension; Table S4. SSR primers used in this study [42,43].

## Sample Availability

Samples of the compounds are not available from the authors.
