# Supplementary material for: Metabolite Profiling of Gardenia jasminoides Ellis In Vitro Cultures with Different Levels of Differentiation
Source: Molecules. 2022 Dec 14;27(24):8906. doi: 10.3390/molecules27248906 (PMC9784620; doi:10.3390/molecules27248906)
Supplement: Supplementary file 1 [file molecules-27-08906-s001.zip › molecules-2084975-supplementary.pdf]

Article

Metabolite Profiling of *Gardenia jasminoides* Ellis. In Vitro Cultures with Different Levels of Differentiation

Gergana Krasteva [1], Strahil Berkov [2], Atanas Pavlov [1,3] and Vasil Georgiev [1,*]

[1] Laboratory of Cell Biosystems, Institute of Microbiology, Bulgarian Academy of Sciences, 139 Ruski Blvd., 4000 Plovdiv, Bulgaria
[2] Institute of Biodiversity and Ecosystem Research, Bulgarian Academy of Sciences, 23 Acad. G. Bonchev, 1113 Sofia, Bulgaria
[3] Department of Analytical Chemistry and Physical Chemistry, Technological Faculty, University of Food Technologies, 4002 Plovdiv, Bulgaria
* Correspondence: vasgeorgiev@gmail.com

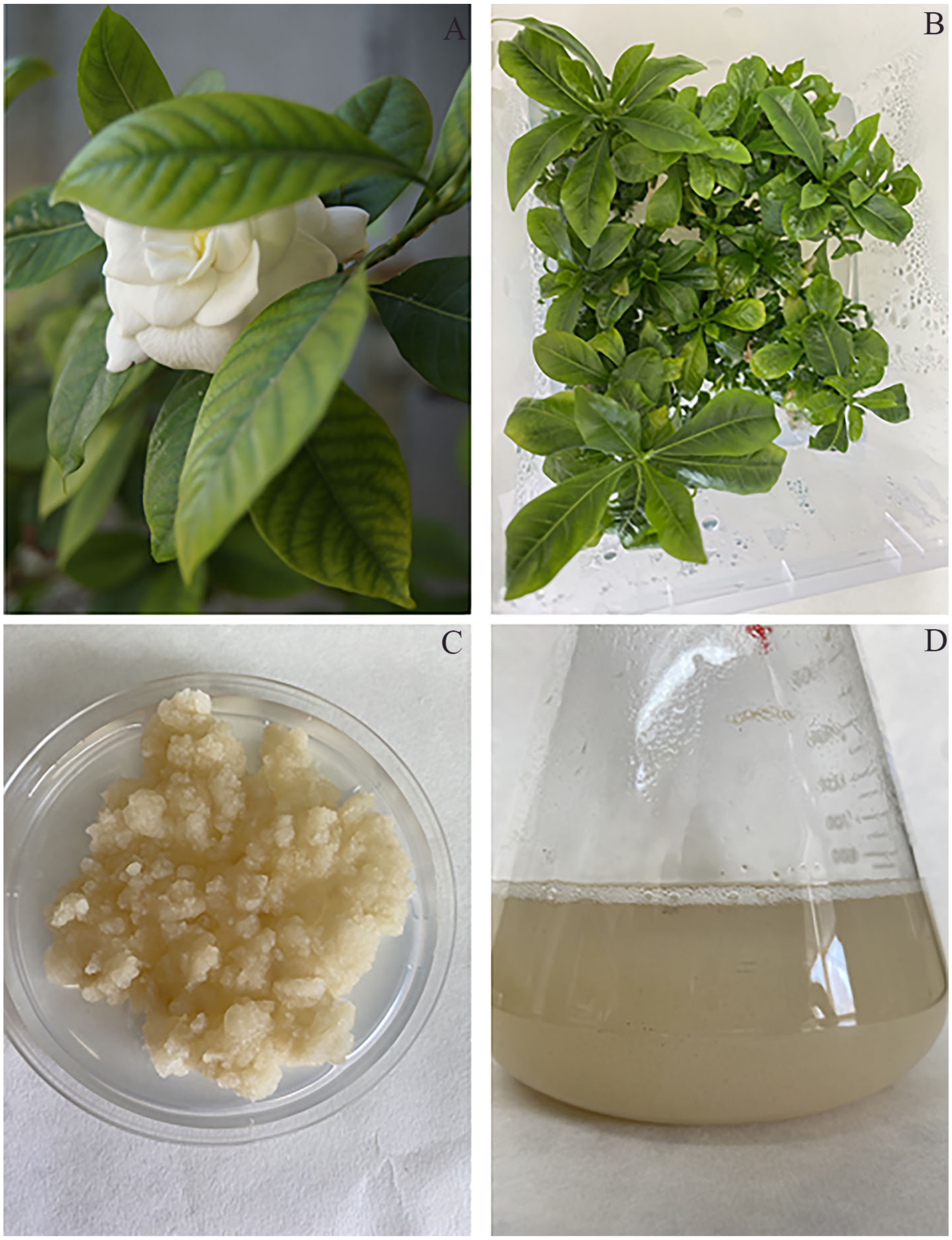

Figure S1. *G. jasminoides* plant (A) and 21-day-old in vitro shoots (B), callus (C), and cell suspension (D) cultures.

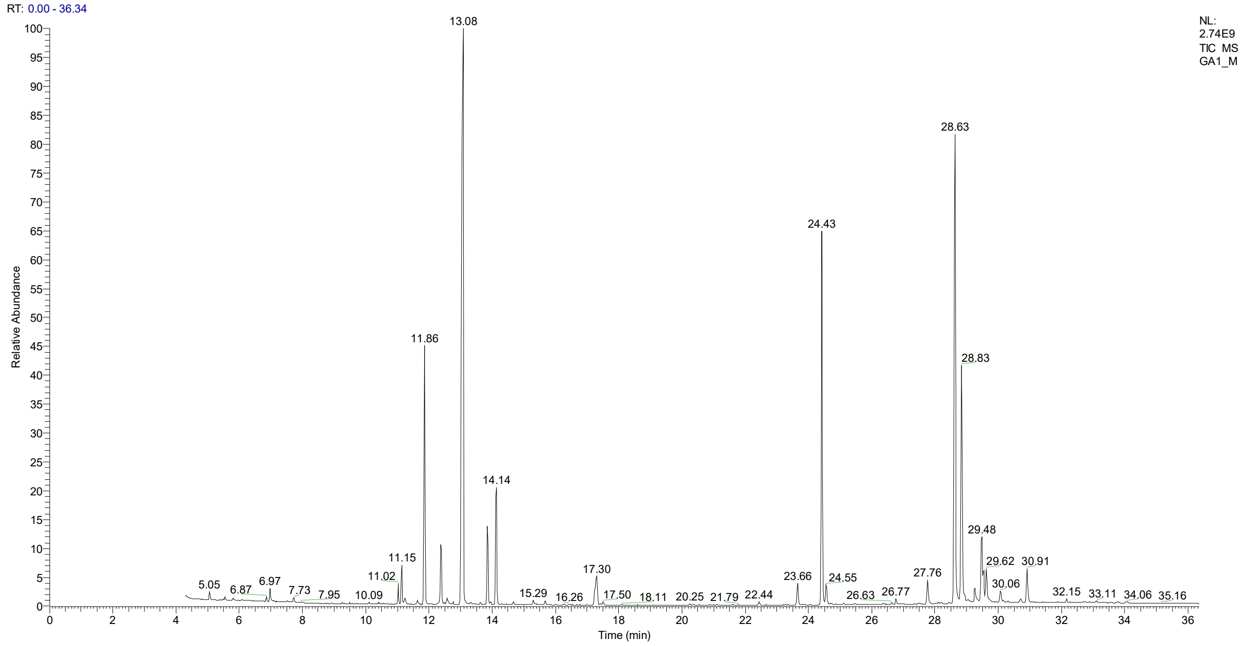

Figure S2. GC/MS chromatogram of extract of *G. jasminoides* plant leaves

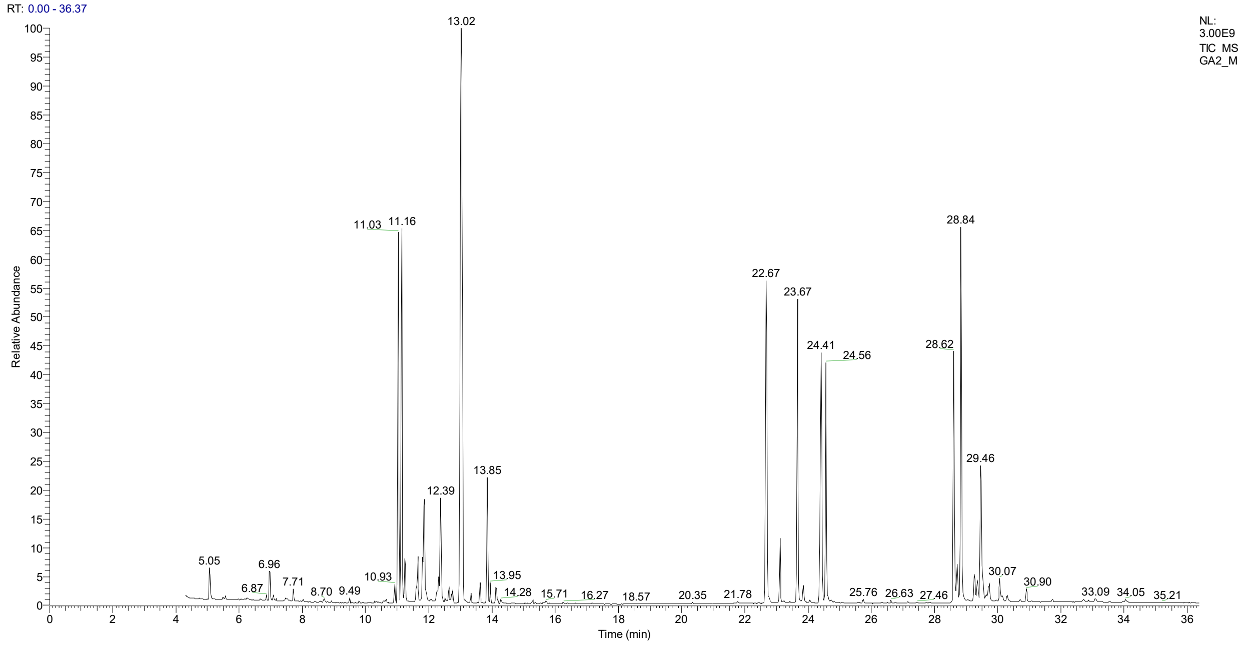

Figure S3. GC/MS chromatogram of extract of *G. jasminoides* shoots culture

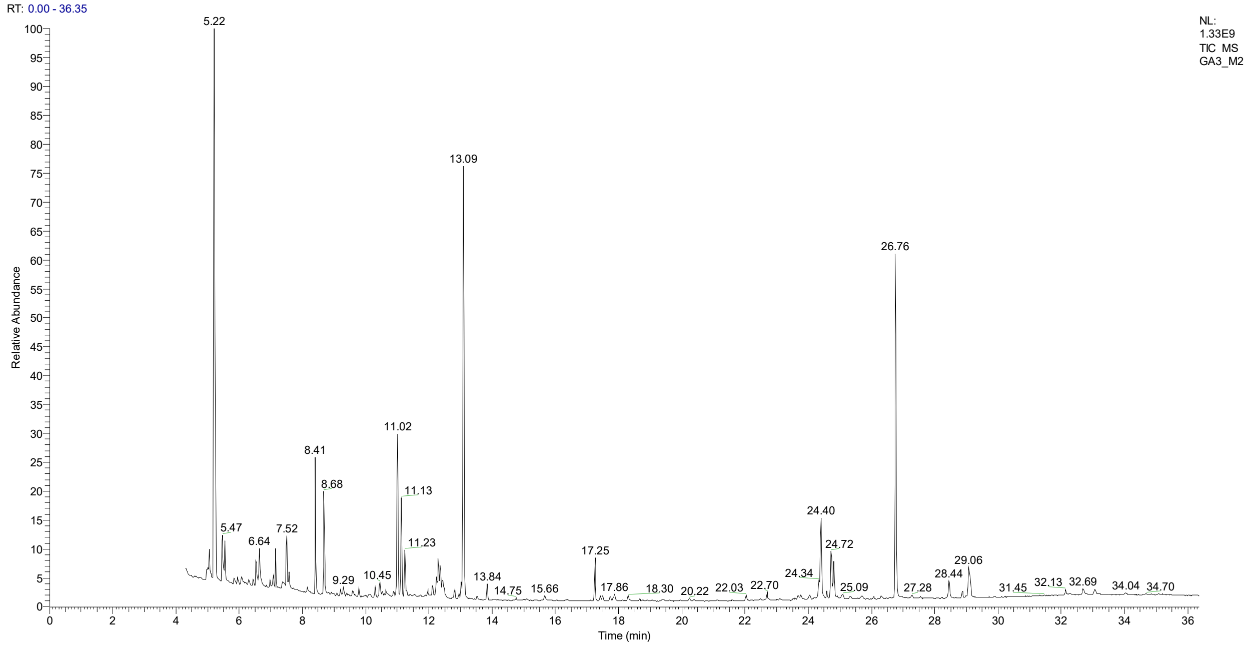

Figure S4. GC/MS chromatogram of extract of *G. jasminoides* callus culture

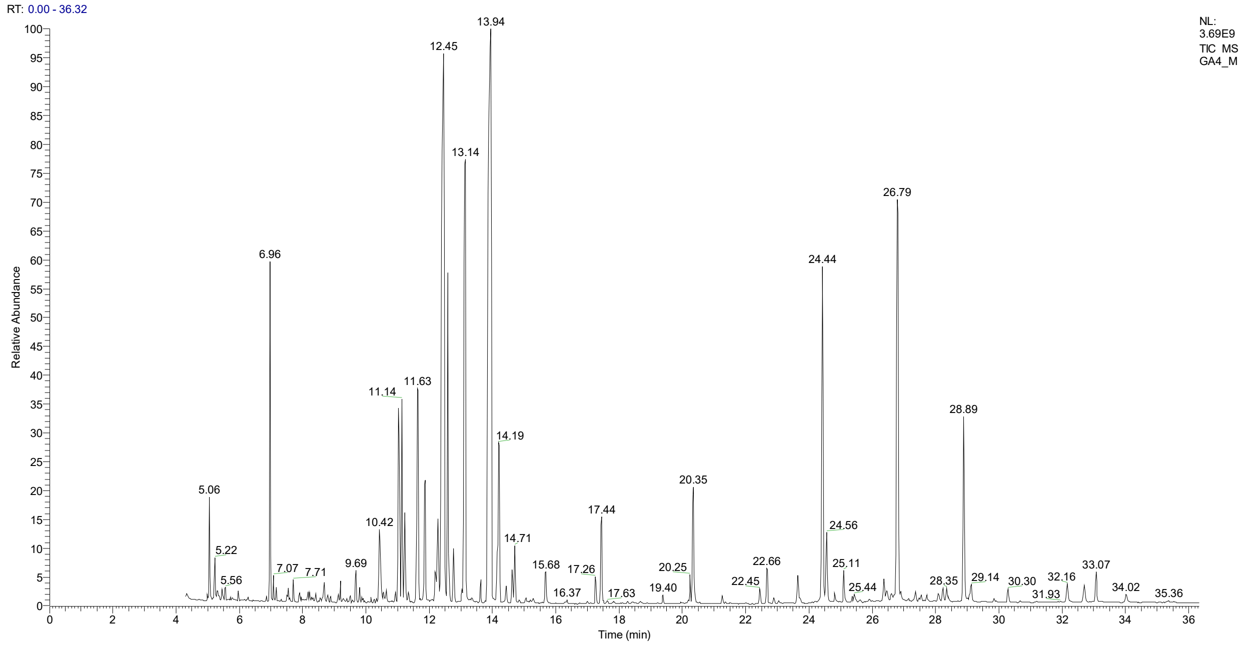

Figure S5. GC/MS chromatogram of extract of *G. jasminoides* cell suspension culture

Table S1. Metabolites identified in extracts of *G. jasminoides* plant leaves and *in vitro* grown shoots, callus and cell suspension by GC/MS.

Compounds	RI*	Plant	Shoots	Callus	Cell suspension
Hydrocarbons fatty alcohols and fatty acids					
Pentane	1493	6.03±0.15	13.06±0.60	24.19±2.61	-
Dodecanol	1564	318.89±24.96	1109.92±20.01	1098.40±29.83	
Hexane	1608	7.51±0.62	15.37±1.54	11.11±1.07	-
Heptane	1701	-	4.52±0.52	-	15.70±1.14
Heptane, branched (Hydrocarbone)	1710	-	3.54±0.49	-	-
Tetradecanoic acid (methyl ester C14:0)	1736	-	14.66±1.61	-	-
Octadecane, 2-methyl	1743	1.29±0.47	2.03±0.50	-	-
1-Tetradecanol	1759	6.21±0.92	41.86±3.14	63.30±5.13	-
Octadecane	1802	11.81±1.76	25.54±4.36	22.77±4.37	-
Hexadecanoic acid (methyl ester , Palmitic acid, C16:0)	1926	308.58±15.86	1156.83±100.33	448.01±45.46	246.23±23.14
1-Hexadecanol	1954	4.12±0.84	32.94±4.67	56.53±8.50	39.06±6.44
Eicosane	1999	8.27±1.16	19.45±2.71	13.77±1.82	19.76±3.00
Hexadecanoic acid (C16:0 TMS)	2041	18.05±3.10	38.08±4.10	15.38±3.38	201.18±9.48
Octadecadienoic acid (methyl ester,Linoleic acid, C18:2)	2095	73.97±7.28	854.01±49.45	63.13±6.74	
Octadecenoic acid (methyl ester, Oleic acid, C18:1)	2100	47.26±7.65	405.01±30.69	50.11±9.13	-
Octadecanoic acid (methyl ester, Stearic acid, C18:0)	2126	120.23±30.41	250.55±47.33	157.27±43.14	57.40±10.04
1- Octadecanol	21504	6.38±1.44	23.58±1.66	-	-
Docosane	2197	6.54±1.49	13.73±8.21	8.81±2.21	15.91±6.96
Eicosanoic acid (methyl ester, Arachidic acid, C20:0)	2331	15.72±9.61	26.58±9.40	-	-
Tetracosane	2398	5.88±3.63	8.55±1.57	-	-
1-Docosanol	2543	-	2.57±0.47	-	-
Hexadecanoylglycerol	2578	16.69±4.93	27.14±6.11	-	182.67±31.91
Hexacosane	2596	2.63±0.36	6.40±0.45	-	-
2-Hydroxydocosanoic acid (methyl ester)	2704	6.42±2.95	17.34±6.07	-	27.42±6.17
1-Tetracosanol	2739	-	2.48±0.64	-	-
1-Monooctadecanoylglycerol	2771	2.66±1.53	12.41±4.57	-	150.07±40.43
Octacosane	2795	-	5.61±1.53	-	-
2-Hydroxytricosanoic acid	2801	1.05±0.85	2.15±1.19	-	-
2-Hydroxy-tetracosanoic acid	2899	-	14.40±4.15	-	18.02±3.80
1-Hexacosanol	2935	0.58±0.46	1.13±0.57	-	-
2-Hydroxy-pentacosanoic acid	2997	-	1.67±0.65	-	-
2-Hydroxy-hexacosanoic acid	3095	2.50±1.57	7.92±2.06	-	-
1-Octacosanol	3131	-	5.89±1.85	-	-

1-Eicosanol	3351	1.84±0.49	1.54±0.73	-	-
Phytosterols					
Campesterol	3241	63.34±3.25	150.95±34.43	860.19±49.61	445.84±47.75
Stigmasterol	3269	60.19±9.06	186.83±14.14	553.94±60.81	418.51±38.40
beta-Sitosterol	3343	210.28±23.20	333.04±32.70	586.72±30.39	324.23±21.68
Terpenes					
Amyrin	3368	-	2.45±0.58	6.77±1.20	-
Organic acids and saccharides					
Phosphoric acid	1267	0.20±0.12	954.20±45.95	191.50±20.18	-
Succinic acid	1315	36.48±6.96	48.49±7.84	225.98±25.81	-
Glyceric acid	1323	56.10±6.42	405.18±34.77	16.07±2.85	73.65±6.59
Fumaric acid	1351	54.48±5.41	-	9.56±1.73	-
Malic acid	1479	470.51±58.59	257.79±31.58	512.38±23.57	855.59±50.95
Erythritol	1490	10.83±1.94	94.60±6.41	28.84±6.52	37.26±3.64
Threonic acid	1547	55.74±5.77	434.88±38.31	-	-
Octanoic acid	1581	218.33±22.95	-	20.94±1.67	-
2,3,4,5-Tetrahydroxypentanoic acid-1,4-lactone	1647	21.13±1.70	-	-	-
Arabitol	1692	26.64±4.91	130.92±24.02	-	-
Ribonic acid	1755	33.07±6.55	34.32±5.71	76.16±5.07	256.57±22.01
Fructose 1	1797	927.73±90.96	9533.62±500.69	10183.61±210.31	37.51±9.44
Fructose 2	1805	1927.25±157.13	8325.78±272.59	12226.25±438.81	1647.84±284.03
Fructose 3	1812	244.29±22.42	1343.23±37.45	9654.63±195.80	1637.65±54.12
Glucose	1885	1440.26±143.64	7266.07±278.35	3636.28±288.35	24495.59±1920.14
Galactose	1927	24428.12±1089.87	11625.88±867.09	178.28±13.00	6253.20±50.94
Myo-Inositol	2084	165.11±23.33	137.39±2.33	26.82±3.64	373.97±17.30
Galactosylglycerol	2363	56.08±4.03	82.36±3.65	12.28±1.96	25.21±0.81
Sucrose	2633	14297.87±659.32	10511.77±489.99	-	4662.04±217.60
Free phenolic acids					
Salicylic acid (Free)	1514	1.10±0.20	-	-	-
2,5-Hydroxybenzoic acid (Free)	1724	1.18±0.17	-	-	-
Protocatechuic acid (Free)	1814	1.25±0.07	-	-	-
Quinic acid (Free)	1851	15.63±0.56	5.44±0.54	2.71±0.27	
3,5-Hydroxybenzoic acid (Free)	2009	11.19±0.47	-	-	-
trans-Ferulic acid (Free)	2095	5.20±0.39	3.64±0.56	8.62±0.33	0.81±0.09
trans-Caffeic acid (Free)	2131	18.77±0.57	1.69±0.24	1.11±0.09	18.57±0.69
3,5-Dimethoxy-4-hydroxycinnamic acid (Free)	2243	-	-	2.68±0.24	1.81±0.22
Bound phenolic acid					

Salicylic acid (Bound)	1515	1.18±0.14	-	-	-
4(p)-Hydroxybenzoic acid (Bound)	1635	-	-	-	12.92±0.28
Vanilic acid (Bound)	1766	4.10±0.23	3.17±0.14	2.03±0.18	11.52±0.37
Protocatechuic acid (Bound)	1814	7.76±0.18	2.72±0.30	-	-
Quinic acid (Bound)	1851	1.63±0.10	0.83±0.07	-	-
Syringic acid (Bound)	1894	3.61±0.15	0.81±0.09	0.61±0.07	-
cis-Ferulic acid (Bound)	1918	11.18±0.44	-	-	-
trans-p-Hydroxycinnamic acid (Bound)	1941	29.57±0.42	13.81±0.08	-	-
cis-Caffeic acid (Bound)	1971	496.05±7.56	88.79±2.11	-	-
Gentisic acid (Bound)	2026	2.25±0.22	0.21±0.08	-	-
3,5-Dimethoxy-4-hydroxycinnamic acid (Bound)	2052	18.61±0.45	26.55±0.49	4.73±0.27	-
trans-Ferulic acid (Bound)	2089	74.35±0.44	45.16±0.47	20.68±0.85	3.27±0.42
trans-Caffeic acid (Bound)	2134	3481.25±183.34	951.22±27.61	14.05±0.32	2.03±0.18
3,5-Dimethoxy-4-hydroxycinnamic acid (t-isomer) (Bound)	2240	133.86±4.96	-	-	7.81±0.12

** - Kovats Indexes (RI) recorded with standard n-hydrocarbon calibration mixture according to Materials and Methods*

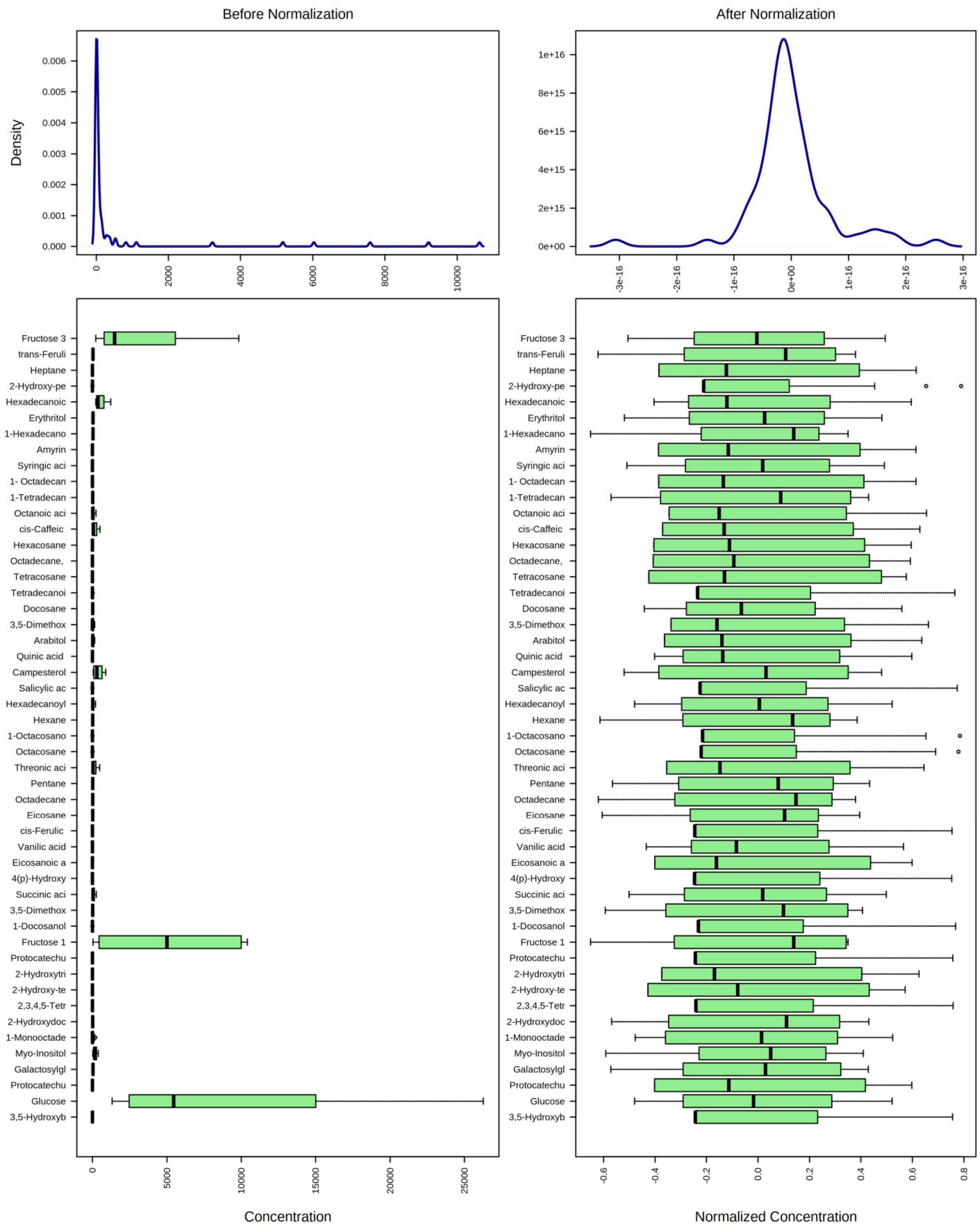

Figure S6. GC/MS data before and after normalization (applied normalization factors = 1, Log 10 data transformation).

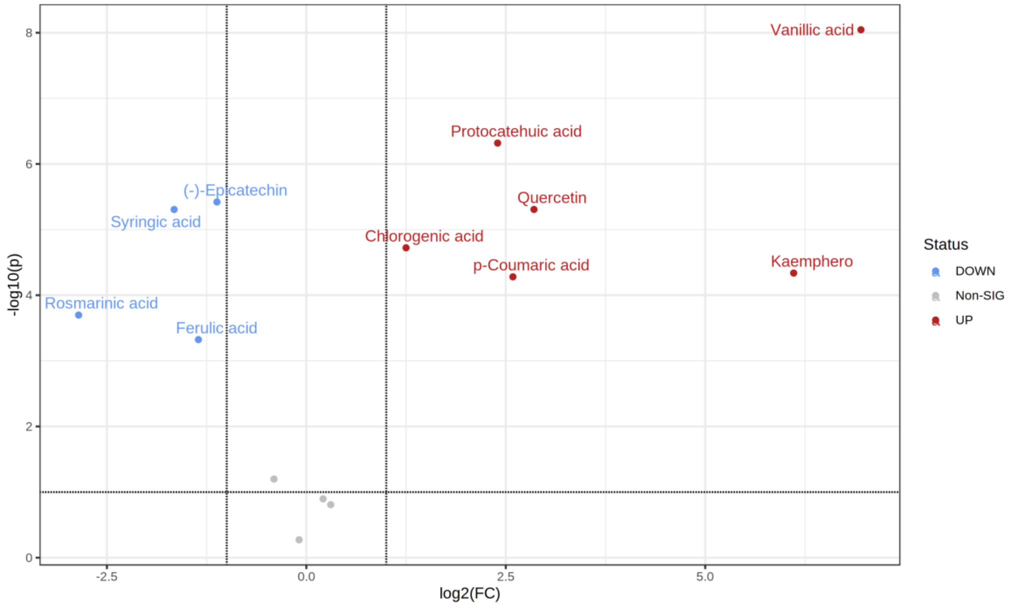

Figure S7. Volcano plot of HPLC data for phenolics, found in *G. jasminoides in vitro* shoot culture in comparison with the same, found in plant leaves. Volcano plot was built by using Fold change (FC) threshold of 2.0 and P-value threshold of 0.01. Volcano plot outline the significant differences in concentrations of compounds found in higher (red) and lower (blue) concentrations in shoots in comparison with the plant leaves.

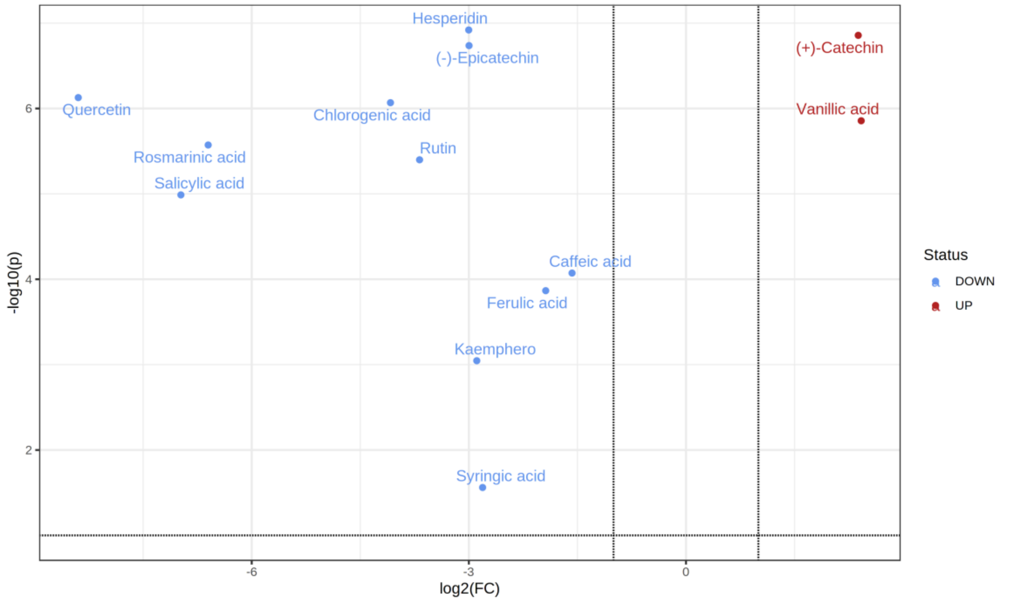

Figure S8. Volcano plot of HPLC data for phenolics, found in *G. jasminoides in vitro* callus culture in comparison with the same, found in plant leaves. Volcano plot was built by using Fold change (FC) threshold of 2.0 and P-value threshold of 0.01. Volcano plot outline the significant differences in concentrations of compounds found in higher (red) and lower (blue) concentrations in callus in comparison with the plant leaves.

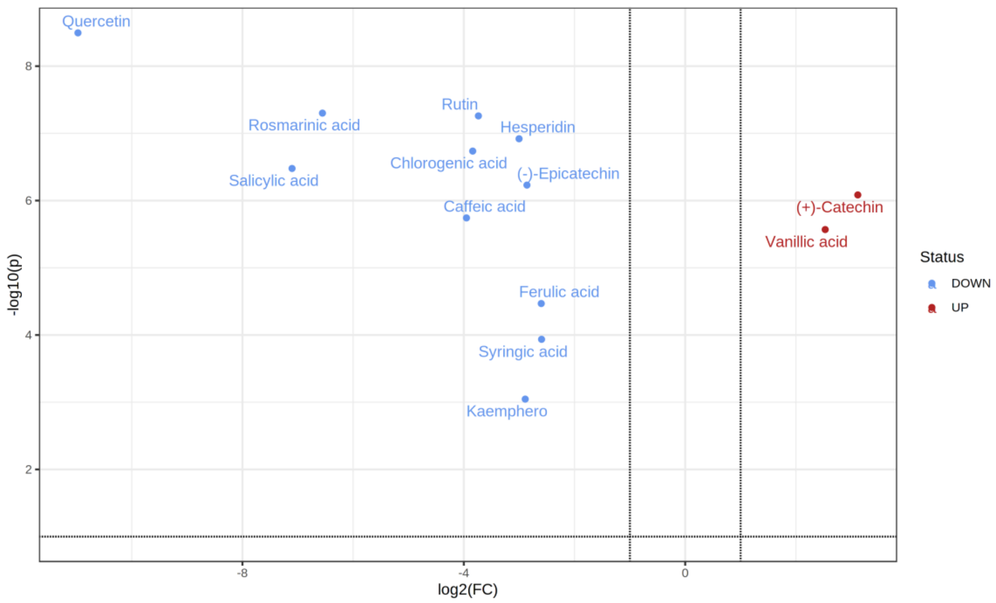

Figure S9. Volcano plot of HPLC data for phenolics, found in *G. jasminoides in vitro* cell suspension culture in comparison with the same, found in plant leaves. Volcano plot was built by using Fold change (FC) threshold of 2.0 and P-value threshold of 0.01. Volcano plot outline the significant differences in concentrations of compounds found in in higher (red) and lower (blue) concentrations in cell suspension in comparison with the plant leaves.

Table S2. Pearson correlation coefficients (r) between observed antioxidant activities (DPPH, TEAC, FRAP, and CUPRAC) and the concentrations of phenolic compounds, detected in extracts of *G. jasminoides* plant leaves and *in vitro* grown shoots, callus, and cell suspension.

	(+)-Catechin	Caffeic acid	DPPH	Chlorogenic acid	Quercetin	Salicylic acid	Rutin	CUPRAC	Hesperidin	TEAC	FRAP	(-)-Epicatechin	Rosmarinic acid	Syringic acid	Ferulic acid	Vanillic acid	Kaemphero	Protocatehuic acid	p-Coumaric acid
**(+)-Catechin**	1	-0.93911	-0.93722	-0.95638	-0.98349	-0.98235	-0.97981	-0.97449	-0.9729	-0.95769	-0.94778	-0.9233	-0.90953	-0.70575	-0.85385	-0.19374	-0.78801	-0.5667	-0.56468
**Caffeic acid**	-0.93911	1	0.77524	0.83383	0.92375	0.863	0.86368	0.86064	0.84764	0.80479	0.78549	0.78185	0.77904	0.54674	0.81199	0.20898	0.71936	0.53328	0.53405
**DPPH**	-0.93722	0.77524	1	0.98922	0.95073	0.9777	0.98069	0.94652	0.96004	0.979	0.98706	0.89853	0.86989	0.70376	0.71352	0.30793	0.85306	0.65829	0.65581
**Chlorogenic acid**	-0.95638	0.83383	0.98922	1	0.98061	0.97687	0.98708	0.94595	0.95152	0.96443	0.97264	0.87073	0.8409	0.67055	0.70655	0.38224	0.89848	0.72046	0.71854
**Quercetin**	-0.98349	0.92375	0.95073	0.98061	1	0.96984	0.97827	0.94358	0.94384	0.93739	0.9375	0.85812	0.83561	0.63463	0.75204	0.36443	0.88365	0.7045	0.70248
**Salicylic acid**	-0.98235	0.863	0.9777	0.97687	0.96984	1	0.99491	0.98209	0.99205	0.98987	0.98681	0.94895	0.93062	0.76395	0.83374	0.18959	0.79606	0.56848	0.56393
**Rutin**	-0.97981	0.86368	0.98069	0.98708	0.97827	0.99491	1	0.97922	0.98616	0.98539	0.9864	0.9271	0.90313	0.73371	0.80371	0.25039	0.83163	0.61944	0.61288
**CUPRAC**	-0.97449	0.86064	0.94652	0.94595	0.94358	0.98209	0.97922	1	0.98623	0.98491	0.97658	0.96609	0.94952	0.79782	0.87775	0.089604	0.72192	0.47858	0.47374
**Hesperidin**	-0.9729	0.84764	0.96004	0.95152	0.94384	0.99205	0.98616	0.98623	1	0.99079	0.98428	0.97265	0.95777	0.78698	0.87547	0.09277	0.73361	0.4871	0.48105
**TEAC**	-0.95769	0.80479	0.979	0.96443	0.93739	0.98987	0.98539	0.98491	0.99079	1	0.99725	0.96576	0.94607	0.79488	0.83126	0.13254	0.75599	0.51655	0.51481
**FRAP**	-0.94778	0.78549	0.98706	0.97264	0.9375	0.98681	0.9864	0.97658	0.98428	0.99725	1	0.949	0.92413	0.7729	0.7978	0.18107	0.78385	0.55619	0.55139
**(-)-Epicatec**	-0.9233	0.78185	0.89853	0.87073	0.85812	0.94895	0.9271	0.96609	0.97265	0.96576	0.949	1	0.9963	0.85355	0.93071	-0.11987	0.57267	0.28795	0.28762

**hin**																			
**Rosmarinic acid**	-0.90953	0.77904	0.86989	0.8409	0.83561	0.93062	0.90313	0.94952	0.95777	0.94607	0.92413	0.9963	1	0.86198	0.94541	-0.17418	0.52585	0.23459	0.23894
**Syringic acid**	-0.70575	0.54674	0.70376	0.67055	0.63463	0.76395	0.73371	0.79782	0.78698	0.79488	0.7729	0.85355	0.86198	1	0.8136	-0.24735	0.35543	0.094695	0.09454
**Ferulic acid**	-0.85385	0.81199	0.71352	0.70655	0.75204	0.83374	0.80371	0.87775	0.87547	0.83126	0.7978	0.93071	0.94541	0.8136	1	-0.32249	0.37317	0.073781	0.069177
**Vanillic acid**	-0.19374	0.20898	0.30793	0.38224	0.36443	0.18959	0.25039	0.089604	0.09277	0.13254	0.18107	-0.11987	-0.17418	-0.24735	-0.32249	1	0.74135	0.91435	0.91171
**Kaemphero**	-0.78801	0.71936	0.85306	0.89848	0.88365	0.79606	0.83163	0.72192	0.73361	0.75599	0.78385	0.57267	0.52585	0.35543	0.37317	0.74135	1	0.94774	0.94287
**Protocatehuic acid**	-0.5667	0.53328	0.65829	0.72046	0.7045	0.56848	0.61944	0.47858	0.4871	0.51655	0.55619	0.28795	0.23459	0.094695	0.073781	0.91435	0.94774	1	0.99434
**p-Coumaric acid**	-0.56468	0.53405	0.65581	0.71854	0.70248	0.56393	0.61288	0.47374	0.48105	0.51481	0.55139	0.28762	0.23894	0.09454	0.069177	0.91171	0.94287	0.99434	1

Table S3. P-values of calculated Pearson correlations between observed antioxidant activities (DPPH, TEAC, FRAP, and CUPRAC) and the concentrations of phenolic compounds, detected in extracts of *G. jasminoides* plant leaves and *in vitro* grown shoots, callus, and cell suspension.

	(+)-Catechin	Caffeic acid	DPPH	Chlorogenic acid	Quercetin	Salicylic acid	Rutin	CUPRAC	Hesperidin	TEAC	FRAP	(-)-Epicatechin	Rosmarinic acid	Syringic acid	Ferulic acid	Vanillic acid	Kaemphero	Protocatehuic acid	p-Coumaric acid
(+)-Catechin	NA	5.95E-06	6.91E-06	1.16E-06	9.41E-09	1.31E-08	2.56E-08	8.16E-08	1.10E-07	9.94E-07	2.80E-06	1.84E-05	4.09E-05	0.010331	0.00040871	0.5463	0.0023331	0.0547	0.055768
Caffeic acid	5.95E-06	NA	0.0030537	0.00074953	1.78E-05	0.00030067	0.00029356	0.00032604	0.00049776	0.001592	0.0024636	0.0026624	0.0028235	0.065846	0.0013366	0.5145	0.0083616	0.074186	0.073692
DPPH	6.91E-06	0.0030537	NA	1.13E-09	2.10E-06	4.18E-08	2.05E-08	3.15E-06	7.50E-07	3.11E-08	2.80E-09	7.13E-05	0.00023507	0.010645	0.0091684	0.3302	0.00041925	0.019944	0.020581
Chlorogenic acid	1.16E-06	0.00074953	1.13E-09	NA	2.09E-08	5.02E-08	2.78E-09	3.32E-06	1.94E-06	4.23E-07	1.15E-07	0.00022789	0.00061066	0.017008	0.010206	0.22011	7.14E-05	0.0082156	0.0084712
Quercetin	9.41E-09	1.78E-05	2.10E-06	2.09E-08	NA	1.87E-07	3.68E-08	4.09E-06	4.00E-06	6.82E-06	6.76E-06	0.00035503	0.0007124	0.026635	0.0047832	0.24416	0.00013773	0.010527	0.010849
Salicylic acid	1.31E-08	0.00030067	4.18E-08	5.02E-08	1.87E-07	NA	2.68E-11	1.41E-08	2.47E-10	8.26E-10	3.07E-09	2.51E-06	1.13E-05	0.0038219	0.0007515	0.55508	0.0019507	0.053777	0.056163
Rutin	2.56E-08	0.00029356	2.05E-08	2.78E-09	3.68E-08	2.68E-11	NA	2.95E-08	3.91E-09	5.12E-09	3.59E-09	1.43E-05	5.70E-05	0.0066055	0.0016334	0.43249	0.00079731	0.031706	0.034098
CUPRAC	8.16E-08	0.00032604	3.15E-06	3.32E-06	4.09E-06	1.41E-08	2.95E-08	NA	3.81E-09	6.02E-09	5.34E-08	3.34E-07	2.37E-06	0.0018738	0.00017453	0.78183	0.0080261	0.1155	0.11975
Hesperidin	1.10E-07	0.00049776	7.50E-07	1.94E-06	4.00E-06	2.47E-10	3.91E-09	3.81E-09	NA	5.15E-10	7.37E-09	1.15E-07	9.85E-07	0.0023855	0.00019068	0.7743	0.0066161	0.10824	0.11336

**TEAC**	9.94E-07	0.001592	3.11E-08	4.23E-07	6.82E-06	8.26E-10	5.12E-09	6.02E-09	5.15E-10	NA	1.24E-12	3.50E-07	3.28E-06	0.0020034	0.00080556	0.68134	0.0044467	0.085531	0.086777
**FRAP**	2.80E-06	0.0024636	2.80E-09	1.15E-07	6.76E-06	3.07E-09	3.59E-09	5.34E-08	7.37E-09	1.24E-12	NA	2.49E-06	1.74E-05	0.0032023	0.0018744	0.57332	0.0025515	0.060391	0.063117
**(-)-Epicatechin**	1.84E-05	0.0026624	7.13E-05	0.00022789	0.00035503	2.51E-06	1.43E-05	3.34E-07	1.15E-07	3.50E-07	2.49E-06	NA	5.43E-12	0.00041277	1.12E-05	0.71058	0.051645	0.3641	0.36467
**Rosmarinic acid**	4.09E-05	0.0028235	0.00023507	0.00061066	0.0007124	1.13E-05	5.70E-05	2.37E-06	9.85E-07	3.28E-06	1.74E-05	5.43E-12	NA	0.00031143	3.48E-06	0.58822	0.07909	0.46301	0.45451
**Syringic acid**	0.010331	0.065846	0.010645	0.017008	0.026635	0.0038219	0.0066055	0.0018738	0.0023855	0.0020034	0.0032023	0.00041277	0.00031143	NA	0.001284	0.43829	0.25688	0.76972	0.77009
**Ferulic acid**	0.00040871	0.0013366	0.0091684	0.010206	0.0047832	0.0007515	0.0016334	0.00017453	0.00019068	0.00080556	0.0018744	1.12E-05	3.48E-06	0.001284	NA	0.30662	0.23217	0.81974	0.83084
**Vanillic acid**	0.5463	0.5145	0.3302	0.22011	0.24416	0.55508	0.43249	0.78183	0.7743	0.68134	0.57332	0.71058	0.58822	0.43829	0.30662	NA	0.0057917	3.14E-05	3.64E-05
**Kaemphero**	0.0023331	0.0083616	0.00041925	7.14E-05	0.00013773	0.0019507	0.00079731	0.0080261	0.0066161	0.0044467	0.0025515	0.051645	0.07909	0.25688	0.23217	0.0057917	NA	2.81E-06	4.35E-06
**Protocatechuic acid**	0.0547	0.074186	0.019944	0.0082156	0.010527	0.053777	0.031706	0.1155	0.10824	0.085531	0.060391	0.3641	0.46301	0.76972	0.81974	3.14E-05	2.81E-06	NA	4.53E-11
**p-Coumaric acid**	0.055768	0.073692	0.020581	0.0084712	0.010849	0.056163	0.034098	0.11975	0.11336	0.086777	0.063117	0.36467	0.45451	0.77009	0.83084	3.64E-05	4.35E-06	4.53E-11	NA

Table S4. SSR primers used in this study.

Locus	Forward Primer	Reverse Primer	GenBank accession #	Reference
GJ02	GGCTCTACAATCTGATTATCTT	ACCTCTAGCAATTCTCCAT	JQ750621	[42]
GJ03	CCTTTCTACCTCCTCCATA	ATCTGACAAGTTCCACCAA	JQ750622	[42]
GJ04	GTCCAACTATCCATAAACAT	AGAAGAAAGAAGGAAACAGA	JQ750623	[42]
GJ08	GGAGCTGAGACTAAAGTAAG	ATCCAGAATCTAAAGCAGT	JQ750627	[42]
GJ09	CGGACCCAGTTCGAGAAGC	ATCCATCGCCTGAGCAACC	JQ750628	[42]
GJ10	TCACCTTTATCACTACCAT	GTTGACAAGTGTTGAGAATA	JQ750629	[42]
GJ16	ATGGAATATCATTTGAGCT	GTAGACGATGTCAGAAACC	JQ750635	[42]
GJ17	GAGATTGGAAATATGAACAC	CAACTCTAGGAACAAGGTA	JQ750636	[42]
eGJ010	GAGGTGTTTGCCATCCTGGA	TGTTCCTCCATTTGCTGCCA	KM279434	[43]
eGJ118	CAACACCTTTGCTCGACTGC	GTCAGCACTCCACAGCATCT	KM279449	[43]
eGJ144	TGAGCTGATGCACTCACAGA	CCTGGACCTGGAGAAAGACG	KM279455	[43]
